# Imaging of infantile hemangiomas: a pictorial essay

**DOI:** 10.1590/0100-3984.2025.0041-en

**Published:** 2025-07-10

**Authors:** Laura Bosi Gil, Carlos Eduardo Nedel, Juliana Gonçalves Silveira, Ana Paula VFB Sperb, Mariane Cibelle Barreto da Silva Barros, Bárbara Limberger Nedel

**Affiliations:** 1 Hospital Moinhos de Vento, Porto Alegre, RS, Brazil; 2 Hospital Femina, Porto Alegre RS, Brazil

**Keywords:** Hemangioma, Hemangioma, capillary, Infant, Ultrasonography, Magnetic resonance imaging, Hemangioma, Hemangioma capilar, Lactente, Ultrassonografia, Ressonância magnética

## Abstract

Infantile hemangioma (IH) is a common benign vascular neoplasm with a characteristic pattern of progression: at birth, it is not fully developed; in the first days or weeks of life, it grows; and its growth peaks at around one year of age, after which there is spontaneous regression. Although most IHs are superficial and therefore obvious on physical examination, they can also affect deeper planes or other organs, in which case they are best assessed with imaging examinations. Methods such as ultrasound and magnetic resonance imaging can help differentiate IHs from vascular malformations, other benign tumors, and malignant tumors. The aim of this pictorial essay is to demonstrate the various presentations of IHs through illustrative cases, with an emphasis on imaging findings.

## Abstract (pt)

O hemangioma infantil (HI) é uma neoplasia vascular benigna comum, com padrão evolutivo característico: ao nascimento não é completamente desenvolvido, e após dias ou semanas de vida cresce, atingindo seu pico por volta de um ano de idade, o qual é seguido por regressão espontânea. Os HIs são geralmente superficiais e, portanto, óbvios ao exame físico, porém também podem acometer planos mais profundos ou outros órgãos, sendo nestes casos mais bem avaliados com exames de imagem. Métodos como ultrassonografia e ressonância magnética podem ajudar a diferenciar os HIs de malformações vasculares e outros tumores benignos ou malignos. Este ensaio iconográfico tem como objetivo demonstrar as diversas apresentações dos HIs por meio de casos ilustrativos, com ênfase nos achados de imagem.

## INTRODUCTION

Infantile hemangioma (IH) is the most common tumor in childhood, with a reported incidence ranging from 2% to 10%^(1^,^2)^. Its etiopathogenesis involves dysregulation in the processes of vasculogenesis and angiogenesis; the risk factors include female sex, low birth weight, prematurity, multiple pregnancies, progesterone therapy during pregnancy, and family history^(3^,^4)^.

An IH is a benign vascular neoplasm with a characteristic progression: at birth it is not fully developed, and after days or weeks it goes through the angiogenic proliferative phase, during which its size and vascularization increase. That phase is most pronounced in the first months of life, peaking at around 12 months of age. The IH then goes through the involution phase, with spontaneous regression at 12-48 months of age. It is estimated that 20-50% of patients have skin remnants after involution^(5^,^6)^.

Although IHs are typically superficial and evident on physical examination, they can be subcutaneous, affecting deep planes or other organs, requiring evaluation by imaging examination. In addition, imaging findings can help differentiate IH from vascular malformations and other tumors^(6^,^7)^.

Doppler ultrasound is the initial method of choice, particularly for superficial or subcutaneous IH, with characteristics that vary according to the disease phase. During the proliferative phase, an IH manifests as a solid, predominantly hypoechoic nodule with well-defined margins, marked vascularization on Doppler flow studies and a predominance of arterial tracings with low resistance indices. In the involution phase, an IH presents as hyperechoic areas due to fatty replacement and a reduction in the intensity and size of the vessels on Doppler flow studies flow studies^(7^,^8)^.

Magnetic resonance imaging (MRI) is often used to evaluate IHs that are more extensive and deeper or when associated syndromes are suspected. In the proliferative phase, an IH presents homogeneous signal intensity that is intermediate on T1-weighted sequences and high on T2-weighted short-tau inversion recovery sequences, containing flow voids and showing intense contrast enhancement, with variable washout. Time-resolved MRI angiography techniques (e.g., time-resolved angiography with interleaved stochastic trajectories and time-resolved imaging of contrast kinetics) allow dynamic evaluation of vascular filling of IHs without the need for a specific time after contrast injection. Apparent diffusion coefficient mapping facilitates the differentiation between IHs and malignant tumors such as sarcomas, which typically have lower apparent diffusion coefficients. During the involution phase, an IH becomes less defined, with a tendency towards heterogeneity, and there is less contrast uptake, reflecting the reduced vascularization^(7^,^8)^.

According to the International Society for the Study of Vascular Anomalies, the classification of IHs can be based on their distribution pattern (focal, multifocal, segmental, or indeterminate) or their depth (cutaneous, purely subcutaneous, mixed, or other), the latter being used for descriptive purposes in this study. The objective of this pictorial essay is to present different IHs through illustrative cases, emphasizing their characteristic imaging findings^(7^-^9)^.

## CUTANEOUS IH

**Figure 1:**
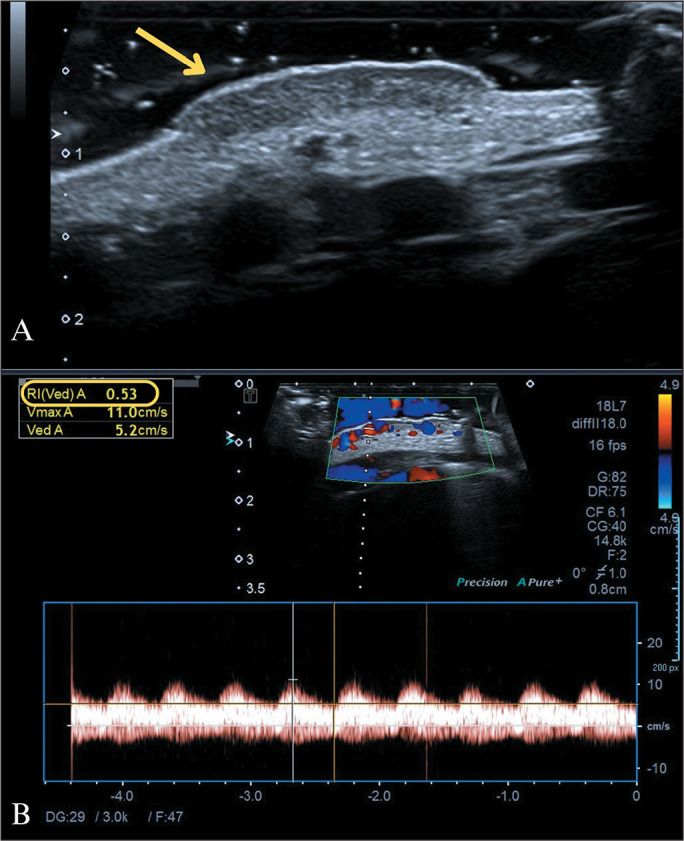
Cutaneous IH. A one-month-old infant with a reddish tumor on the anterior surface of the chest. A: Ultrasound image obtained with a high-frequency transducer, showing a slightly exophytic, hypoechoic, expansile formation restricted to the cutaneous planes, with well-defined margins and lobulated edges. B: Color Doppler showing intense vascularization and an arterial flow pattern with a low (0.53) resistance index (RI). These findings are characteristic of a cutaneous IH.

Cutaneous IH, which accounts for more than half of all cases of IH, presents as an intensely red lesion with raised edges during the proliferative phase and is evident on physical examination ^(5)^ . Cutaneous IH can be well evaluated by ultrasound with a high-frequency transducer (Figure 1), and a gel pad should be used to avoid compression.

## PURELY SUBCUTANEOUS IH

**Figure 2:**
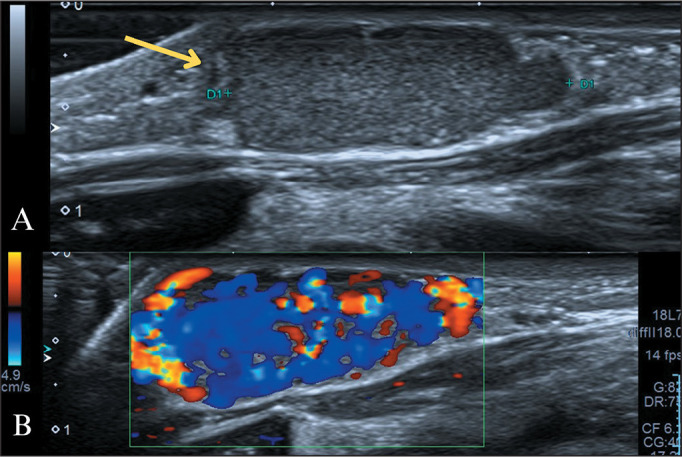
Purely subcutaneous IH. A three-month-old infant with a palpable nodule in the left sternoclavicular region. A: High-frequency ultrasound image showing an expansile, hypoechoic formation restricted to the subcutaneous planes, with well-defined margins. B: Color Doppler showing intense vascularization, which is consistent with purely subcutaneous IH.

A purely subcutaneous IH is completely embedded in the subcutaneous fat, presenting a purplish-blueish hue or even no visible changes on the skin, making its clinical diagnosis challenging^(7)^. Purely subcutaneous IHs are amenable to excellent evaluation by ultrasound with a high-frequency transducer (Figure 2).

## MIXED (CUTANEOUS AND SUBCUTANEOUS) IH

**Figure 3:**
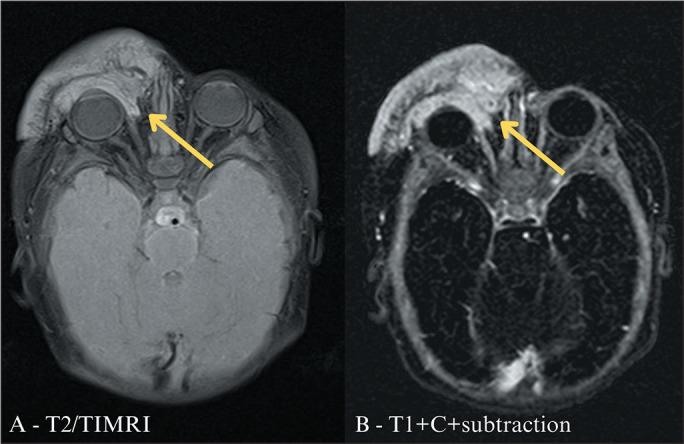
Mixed IH. A two-month-old infant with a reddish bulging/expansile lesion on the right side of the face. MRI scans showing an expansile lesion with the center affecting the cutaneous and subcutaneous planes of the right face, involving the zygomaticomaxillary, preseptal/palpebral, and nasal regions, extending slightly into the medial extraconal postseptal space of the orbit. It was characterized by a predominantly hyperintense signal on T2-weighted/T1-weighted sequences (T2/TIMRI, in A) and intense contrast enhancement on contrast-enhanced T1-weighted sequences with digital subtraction (T1+C+subtraction, in B), findings that are consistent with a mixed IH.

A mixed IH manifests as subcutaneous nodulation with areas of cutaneous involvement, leading to discoloration or reddish coloration of the skin. It usually has a flexible, mobile consistency and can occur in any region of the body ^(7)^ . Figure 3 demonstrates the MRI features of a mixed IH of the face, with segmental distribution, a characteristic that increases the risk of complications and often requires intensive treatment. In addition, segmental lesions are more commonly associated with syndromes, such as the posterior fossa malformation-hemangioma infantile-arterial anomalies-cardiac defects-eye malformation-sternal cleft syndrome and the spinal dysraphism-anogenital anomalies-cutaneous anomalies-renal and urologic anomalies-angioma-lumbosacral localization syndrome^(7)^, the details of which exceed the scope of this study.

## OTHER

### Orbital IH

**Figure 4:**
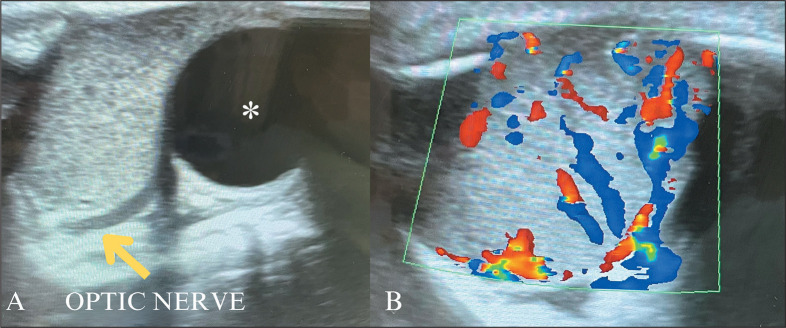
Orbital IH. A two-week-old infant with progressive left-eye proptosis. A: Ultrasound of the orbit, showing an expansile intraorbital lesion, with homogeneous echotexture and well-defined margins, near the globe (asterisk) and the optic nerve (arrow). B: Color Doppler showing signs of intense vascularization, a set of findings that are consistent with orbital IH.

Orbital IH is the most common benign neoplasm in the pediatric orbit and can cause ophthalmologic complications such as proptosis and compression of the optic nerve, which makes its early diagnosis essential. When there is suspicion of an expansile orbital lesion in an infant, ultrasound can be used for the initial evaluation, with the advantage of not requiring anesthesia, and the ultrasound findings of an orbital IH are shown in Figure 4. The gold-standard examination for the evaluation of orbital IH and its relationship with orbital structures, such as the eyeball, optic nerve, intraconal and extraconal planes, and orbital musculature, is MRI. An orbital IH has characteristics similar to those of other IHs in the proliferative phase, with a hyperintense signal on T2-weighted sequences, flow voids, and intense contrast uptake^(10)^.

### Airway IH

**Figure 5:**
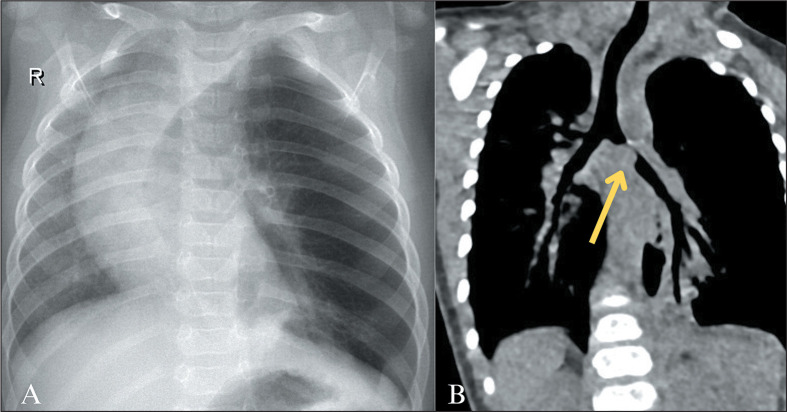
Airway IH. A four-month-old infant with progressive respiratory dysfunction, admitted to the hospital with cough, subcostal retraction, and peripheral oxygen saturation of 95%. Chest X-ray showing asymmetric lung volumes, with hyperexpansion of the left lung and mediastinal shift to the right (R, in A), the initial diagnostic hypotheses being congenital lobar emphysema and foreign body aspiration. Further investigation with unenhanced chest CT (B) showed a 1.0-cm nodular opacity significantly reducing the lumen of the right main bronchus (arrow), and direct endoscopic evaluation was indicated.

**Figure 6:**
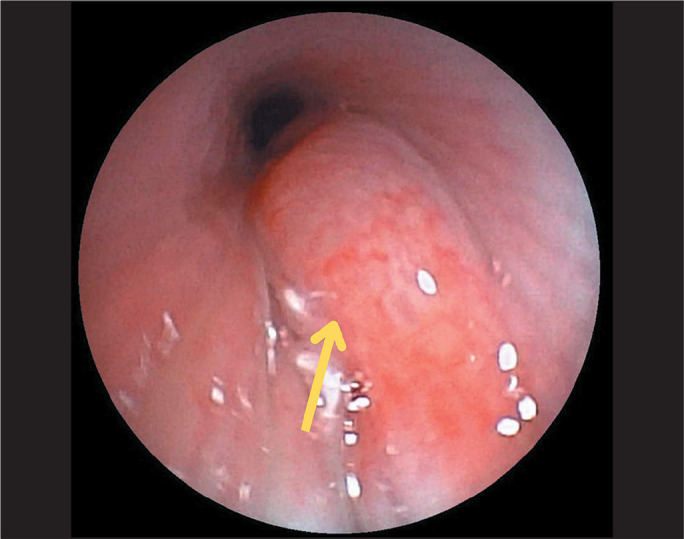
Airway IH. Fiberoptic bronchoscopy image showing a sessile lesion covered by vascularized mucosa, completely obstructing the lumen of the left main bronchus (arrow). For diagnostic and therapeutic purposes, the bronchial lumen was partially opened and the endobronchial tumor was biopsied. After the biopsy, there was significant bleeding, which was contained with cold saline and adrenaline.

**Figure 7:**
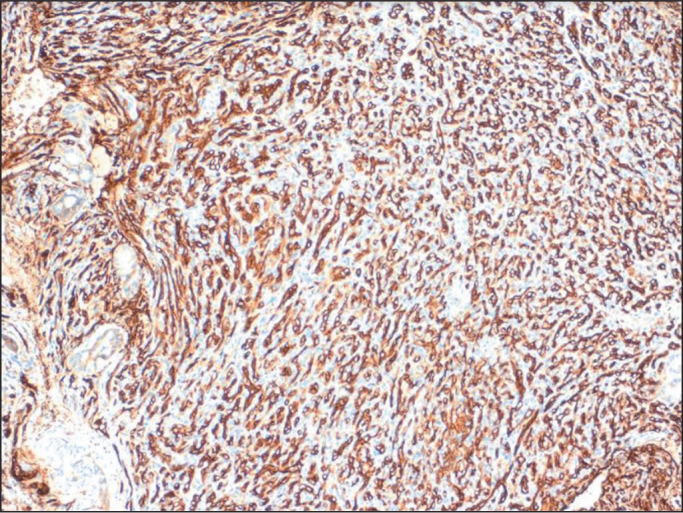
irway IH. Histopathologic findings after biopsy by fiberoptic bronchoscopy demonstrated vascular channels lined by round endothelial cells, with discrete or imperceptible lumens. Photomicrograph demonstrating immunoexpression of the glucose transporter type 1 protein, characterized by intense, diffuse brown staining in the endothelial cells of blood vessels, a finding that helps differentiate IHs from other vascular lesions.

Airway IH is a rare condition that can affect any portion of the airway, with a predilection for the subglottis, and half of all cases also present with cutaneous IH. Up to 90% of infants with an airway IH will present symptoms by six months of age, due to growth in the proliferative phase, which can lead to stridor, respiratory failure, or airway obstruction. The presence of nodules in the airway on imaging tests-computed tomography (CT) or MRI-should raise the possibility of airway IH, with the diagnosis being confirmed by laryngoscopy and bronchoscopy. Biopsy is usually not necessary, however, when there is diagnostic uncertainty, immunohistochemical analysis reveals protein expression of glucose transporter type 1. Figures 5 to 7 demonstrate findings of airway IH by radiology (X-ray and CT), fiberoptic bronchoscopy, and histopathology, respectively.

### Parotid IH

**Figure 8:**
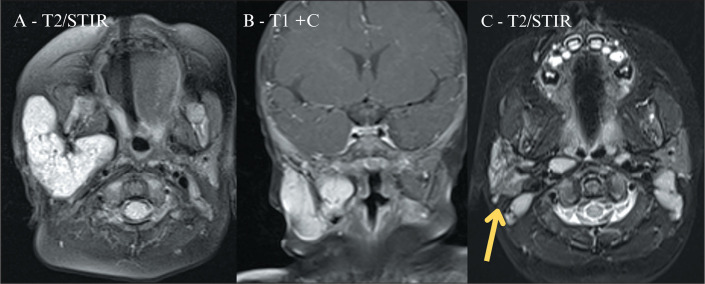
Parotid IH. A three-month-old infant with a progressive volumetric increase in the right cervical region. An outpatient ultrasound evaluation showed an expansile lesion in the cervical region, and MRI was performed to assess its relationship with deep planes. The MRI sequences showed an expansile lesion with lobulated contours, presenting a hyperintense signal on an axial T2-weighted short-tau inversion recovery sequence (T2/STIR, in A), as well as intense enhancement on a coronal T1-weighted gadolinium contrast-enhanced sequence (T1+C, in B), findings consistent with an IH, in the right parotid gland. Oral propranolol was administered, with a satisfactory response on physical examination, confirmed by a follow-up MRI scan (C), which showed a significant reduction in the size of the IH, with heterogeneous signal intensity (arrow), secondary to fatty replacement of the lesion.

Parotid IH is the most common benign tumor of the infantile parotid gland. During the proliferative phase, ultrasound reveals a parotid IH as a lobulated, hypoechoic or hyperechoic nodule that is hypervascular on a Doppler flow study. The MRI findings before and after oral propranolol therapy are shown in Figure 8.

### Hepatic IHs

**Figure 9:**
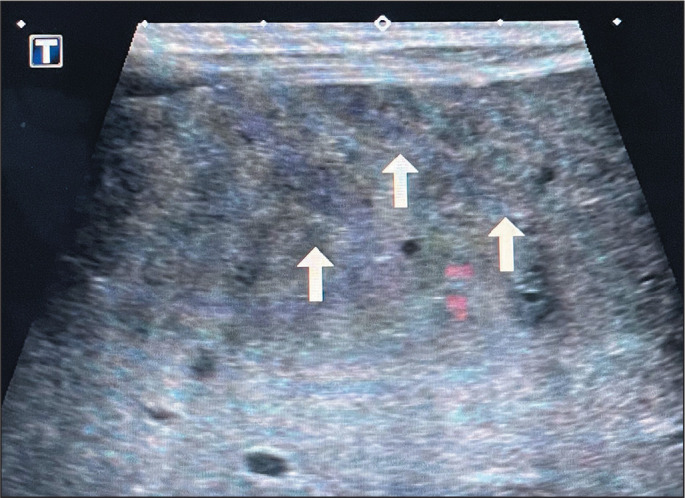
Hepatic IHs. A one-week-old infant with hepatomegaly on the prenatal examination. High-frequency ultrasound showing numerous small hypoechoic nodules in the liver parenchyma (arrows), characteristic of IHs, in contrast with hepatic hemangiomas in adults, which are typically hyperechoic.

**Figure 10:**
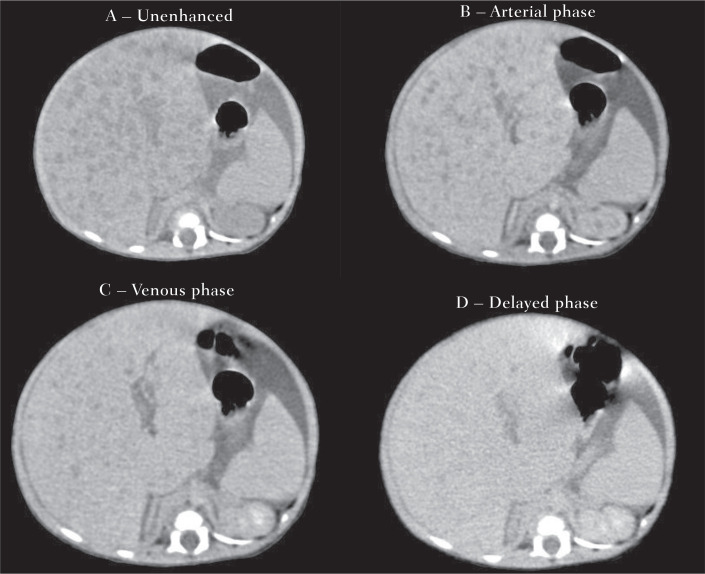
Hepatic IHs. Further CT investigation to evaluate nodules. Multiphasic CT showing an enlarged liver, due to several hypodense nodules that present early peripheral contrast enhancement and gradual, progressive centripetal filling, with a tendency toward homogenization in relation to the hepatic parenchyma in late acquisitions, findings consistent with infantile hepatic hemangiomatosis.

Hepatic IHs, although rare, constitute the majority (approximately 60%) of pediatric liver tumors. They occur before the age of two months and are typically asymptomatic. The presence of five or more cutaneous IHs is associated with a hepatic IH, and investigation by imaging is indicated in such cases^(8)^. Hepatic IHs can be multifocal (multiple small nodules) or diffuse (masses that replace and enlarge the liver). Ultrasound evaluation demonstrates hypoechoic homogeneous nodules-unlike the typically hyperechoic nodules seen in adults with hepatic hemangioma-with variable internal vascularization on Doppler flow studies, as well as increased caliber of the hepatic artery and veins^(8)^. Microbubble contrast demonstrates the pattern of impregnation of the nodules, with early peripheral enhancement and gradual centripetal filling, similar to contrast-enhanced CT and MRI studies. The gold-standard method is MRI, an examination that does not expose patients to ionizing radiation. On MRI, a hepatic IH appears as a predominantly homogeneous lesion that is hypointense on T1-weighted sequences and hyperintense on T2-weighted sequences, with centripetal, progressive gadolinium uptake and a tendency toward homogenization in relation to the hepatic parenchyma in late acquisitions^(8)^. Figures 9 and 10 illustrate hepatic IHs on contrast-enhanced ultrasound and CT examinations, respectively.

## CONCLUSION

Because of its wide availability, together with its ability to characterize the echogenicity and vascularization patterns of superficial lesions, color Doppler ultrasound is the method of choice for evaluating IHs. However, MRI is an essential complementary examination, especially for lesions that are large or deep or when associated syndromes are suspected^(7)^.

The radiologist plays a fundamental role in individualizing the indication of the best method to evaluate lesions suspected of being IHs, as well as in the interpretation of the imaging examinations and differential diagnosis. Adequate knowledge of the imaging characteristics of IHs improves diagnostic accuracy, promoting better clinical outcomes.
